# Behavior of Heat-Denatured Whey:Buttermilk Protein Aggregates during the Yogurt-Making Process and Their Influence on Set-Type Yogurt Properties

**DOI:** 10.3390/foods2040444

**Published:** 2013-09-30

**Authors:** Maxime Saffon, Véronique Richard, Rafael Jiménez-Flores, Sylvie F. Gauthier, Michel Britten, Yves Pouliot

**Affiliations:** 1 STELA Dairy Research Center, Institute of Nutrition and Functional Foods (INAF), Laval University, Quebec City, QC, Canada, G1V 0A6; E-Mails: maxime.saffon.1@ulaval.ca (M.S.); veronique.richard@fsaa.ulaval.ca (V.R.); sylvie.gauthier@fsaa.ulaval.ca (S.F.G.); 2 Dairy Products Technology Center (DPTC), California Polytechnic State University, San Luis Obispo, CA 93405, USA; E-Mail: rjimenez@calpoly.edu; 3 Food Research and Development Center (FRDC), Agriculture and Agri-Food Canada, St-Hyacinthe, QC, Canada, J2S 8E3; E-Mail: Michel.britten@agr.gc.ca

**Keywords:** yogurt, protein aggregates, texture properties, buttermilk

## Abstract

The objective of this study was to assess the impact of using heat-denatured whey:buttermilk protein aggregate in acid-set type yogurt production. Whey and buttermilk (25:75) protein concentrate was adjusted to pH 4.6, heated at 90 °C for 5 min, homogenized and freeze-dried. Set-type yogurts were prepared from skim milk standardized to 15% (w/v) total solids and 4.2% (w/v) protein using different levels of powdered skim milk or freeze-dried protein aggregate. The use of the protein aggregate significantly modified yogurt texture, but did not affect the water-holding capacity of the gel. Confocal laser-scanning microscope images showed the presence of large particles in milk enriched with protein aggregate, which directly affected the homogeneity of the clusters within the protein matrix. Thiol groups were freed during heating of the protein aggregate suspended in water, suggesting that the aggregates could interact with milk proteins during heating.

## 1. Introduction

The use of microparticulated whey proteins (MWP) as a fat replacer in yogurt systems has been investigated. This dry dairy product was developed initially in order to provide means of increasing the retention of whey proteins by casein during cheese curd formation [1,2,3]. It is obtained by heating ultrafiltered whey at 90–95 °C for several minutes with the pH at acidic (4.2–4.6) or neutral (6.2–6.7) values [1,4,5] and spray drying. It is generally accepted that the product does not interact with milk proteins during the cheese-making process and that the micro-particles are only trapped within the casein network. It is still unclear whether MWP acts as active or inert particles in yogurt. Tamine *et al.* [6] described the role of a fat replacer in yogurt as simulating fat globules without interacting with other milk proteins. Sandoval-Castilla *et al.* [7] and Tamine *et al.* [6] concluded that MWP not only becomes part of the protein matrix, but also modifies the microstructure of the acid gel. The gels thus formed are lower in tension and in firmness, but higher in cohesiveness [6,7,8]. The resulting yogurt is also more susceptible to syneresis.

The major drawback associated with MWP is the high water-holding capacity of the micro-particles, which contribute directly to increasing the moisture content of the curd. Saffon *et al.* [9] proposed reducing the water-holding capacity of MWP by combining the whey starting material with buttermilk. They showed that this significantly decreased the water-holding capacity of the resulting micro-particles, as well as modifying their properties [9]. Once viewed as a by-product of butter-making, buttermilk is now considered a valuable product because of its high content in fragments of milk fat globule membrane (MFGM), in addition to phospholipids and whey proteins [10,11]. However, studies by Mistry *et al.*, Raval *et al.*, and Turcot *et al.* indicated that the moisture content of cheese supplemented with buttermilk remained high, due largely to phospholipids [12,13,14,15].

In the present work, less-hydrated aggregates of heat-denatured whey:buttermilk protein were added as a substitute for skim milk powder to skim milk used for yogurt production. The main objective of this study was to understand the behavior of these aggregates during the yogurt-making process. The effect of different levels of skim milk powder substitution (0%–100%) on the textural properties of acid set-type yogurt was tested. The water-holding capacity of the resulting gels, the particle size distribution after heating, the liberation of thiol groups during heating, and the average size of the aggregates were investigated in order to gain better understanding of the texture results.

## 2. Experimental Section

### 2.1. Materials

Fresh whey from mozzarella cheese production and fresh buttermilk were both obtained from a local cheese producer (L’Ancêtre, Bécancour, QC, Canada). Powdered whey permeate was obtained from Maple Leaf Foods International (Toronto, ON, Canada) and powdered skim milk was obtained from Dairytown Products Limited (Sussex, NB, Canada). Pasteurized skim milk was purchased at a local grocery outlet and yogurt starter was obtained from Yogotherm (Saint-Hyacinthe, QC, Canada). Oregon Green 488 was obtained from Life Technologies Corporation (Burlington, ON, Canada). All other reagents were obtained from Fisher Scientific (Ottawa, ON, Canada).

### 2.2. Preparation Heat-Denatured Aggregates of Whey:Buttermilk Protein

Heat-denatured aggregates of whey:buttermilk protein were formed using our method as described previously [9]. Briefly, cheese, whey and buttermilk were skimmed using a pilot-scale milk separator (Alfa-Laval, Uppsala, Sweden) and then concentrated separately by ultrafiltration (UF) through a 5 kDa membrane (Romicon, Koch Membrane Systems, Wilmington, MA, USA) to a final protein concentration of 9.5% (w/v). Concentrates were mixed at a whey:buttermilk protein ratio of 25:75. Mixtures were adjusted to pH 4.6 by slow addition of 6 N HCl and heated from 4–90 °C for 16 min (including ramp time, about 11 min at 90 °C) in a stirred cooker with a steam jacket. The heated liquid was then homogenized three times at 69 MPa using an Emulsiflex C-500 device (Avestin Canada, ON, Canada). The homogenates were freeze-dried in order to obtain powdered whey:buttermilk aggregate (WBAP).

### 2.3. Yogurt Production

Skim milk containing 8.4% (w/v) total solids and 3.6% (w/v) protein was standardized to 15% total solids and 4.2% protein using powdered skim milk and powdered permeate. WBAP was added to replace 0%, 20%, 40%, 60%, 80% or 100% of the added powdered skim milk. The milk thus enriched was stirred for 30 min at 4 °C and then heated to 85 °C in a Stephan cooker with thermostatic control (Sympak France, Lognes, France), with constant stirring for 25 min including ramp time. After cooling to 42 °C in an ice water bath, the milk was inoculated with a commercial yogurt starter (*Streptococcus thermophilus* and *Lactobacillus bulgaricus*) and incubated at 42 °C until a pH of 4.6 was obtained. For each enrichment level, 16 yogurts were analyzed after 18 h at 4 °C, eight were analyzed after 7 days, and eight after 14 days. Yogurts were also prepared in falcon tubes (25 mL). Each production was repeated three times.

### 2.4. Analytical Method

#### 2.4.1. Compositional Analysis

Overall composition was determined using standard methods. Total nitrogen content was determined by the Dumas combustion method using a LECO device (Protein Analyzer method FP-528 Leco Instruments Ltd., Mississauga, ON, Canada) [16]. Nitrogen values were converted into protein values using 6.38 as the nitrogen conversion factor. Fat was determined using the Mojonnier extraction method, and lactose was determined using the phenol-sulfuric acid method [17]. Total solids were obtained by microwave drying (Smart System 5, CEM Corp., Matthews, NC, USA) and ash was measured by incineration in a refractory oven at 550 °C for 20 h [18]. 

#### 2.4.2. Textural Properties

The texture of the set-type yogurts was evaluated by penetration using a TA-XT2 texture analyzer (Texture Technologies Corp., Scarsdale, NY, USA) connected to a computer running the Stable Micro System (Stable Micro Systems Limited, Surrey, UK), with a cylindrical probe (diameter 12.5 mm) and 5 kg load cell. Yogurts were analyzed in their containers (56 cm diameter with yogurt depth of 3.5 cm). The following parameters were set: speed 60 mm/min, distance 20 mm, hold-up time 30 s, force 0.01 Newton (N). Rupture force corresponded to the first peak, firmness to the maximum force, and adhesiveness to the resistance to withdrawal of the penetration probe from the sample. Relaxation (Equation 1) was calculated from the firmness force and force after 30 s of holding. Tests were carried out immediately after moving the samples from the cold room.

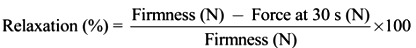
(1)

#### 2.4.3. Water-Holding Capacity of Yogurt

Yogurt prepared in Falcon tubes (25 mL) was centrifuged at 222× *g* for 10 min at 4 °C. The clear supernatant was poured off and weighed. Water-holding capacity (Equation 2) was calculated from the weight of the supernatant and that of the yogurt:

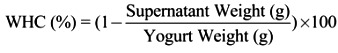
(2)

#### 2.4.4. Milk Particle Size Distribution

Skim milk was enriched with WBAP in the same proportions as for yogurt production and stirred for 30 min at 4 °C before analysis. Particle size distribution before and after heating was measured using a Mastersizer 2000 laser diffraction system (Malvern Instruments, Worcestershire, UK). Samples were shaken by inversion for 1 min and dispersed directly into the recirculating cell until a 12%–19% obscuration value was reached. The dispersant (deionized water) was stirred at 950 rpm and measurements were started after 5 min. Data were expressed as the volume-weight average particle size, also known as the *D*[4,3] value. Five measurements per sample were averaged. The particle size distribution of WBAP before and after homogenization was measured under the same conditions. 

#### 2.4.5. Exposure of Free Thiol Groups during Heating

Exposure of free thiol groups was measured using the method of Ellman [19]. A 250 μL sample of WBAP-enriched skim milk was taken every 5 min during heating and mixed with 2.5 mL of 0.1 M sodium phosphate buffer (containing 1 mM EDTA; pH 8.0) and 0.1 mL of DTNB reagent solution (4 mg in 1 mL of sodium phosphate buffer). Ammonium persulfate (0.2000 ± 0.0050 g) was added after 30 min of incubation at room temperature. Absorbance was measured at 412 nm using a Multiskan Spectrum (Thermo Labsystems, Vantaa, Finland) relative to a blank. The theoretical exposure was calculated as follows (Equation 3):

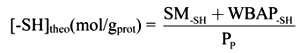
(3)
where [-SH]_theo_ is the concentration of exposed free thiol groups in moles per gram of protein in the sample containing 100% WBAP substitution of the skim milk powder supplement, SM_-SH_ is moles of thiol group contributed by the skim milk, WBAP_-SH_ is moles of thiol group contributed by WBAP, and P_P_ is g of protein in the mixture.

### 2.5. Confocal Laser-Scanning Microscopy

The imaging procedure was adapted from the procedure developed by Hennessy [20] and was carried out using a Nikon C1 laser-scanning confocal microscope at 60× with water objective (Nikon Corporation). Briefly, skim milk with 0% or 100% of the enrichment replaced with WBAP was placed for 11 min in boiling water with constant stirring. After cooling to room temperature, 10 mL aliquots were treated with 1.000 ± 0.050 g of glucano-delta-lactone and then immediately with 3 μL of Oregon Green 488 (0.1 g·L^−1^ in dimethylformamide) to stain proteins and with 3 μL of Nile red (0.01 g·L^−1^ in dimethyl sulfoxide) to stain phospholipids. A 75 μL droplet of the mixture was deposited on a cover slide placed on top of the laser, and images were captured every 15 s for 20 min beginning at 2 min after adding glucono-delta-lactone. Files were transformed into six-frame-per-second videos using ImageJ (U.S. National Institutes of Health, Bethesda, MD, USA; [21]). The videos are available at [22]. Single pictures were taken at the end of the gel formation using the following parameters: resolution = 1024/1024; quality = 13 db; zoom = 2.31×. The dark level was set at 0 in order to limit background noise and to define the clusters more clearly. Images were loaded into ImageJ, transformed to 8-bit, and their threshold was adjusted to cover all the dark areas.

### 2.6. Statistical Analysis

All experiments were performed in triplicate, each value thus representing the mean of three measurements. All data were tested as a factorial experiment with powdered skim milk substitution level × time. Time corresponded to storage time at 4 °C for texture and WHC analysis, and to heating time for particle size distribution and thiol group exposure in the enriched milk. Statistical analysis was carried out using the SAS PROC GLM procedure (SAS Institute Inc., Cary, NC, USA). Mean comparisons were performed using a Duncan post-test. Table 1 and Table 2 summarize the significance of each treatment for each variable. Results were considered significantly different when *p* < 0.05.

**Table 1:** Summary of the significance of the effects of powdered whey:buttermilk aggregate (WBAP) and time on the texture and water-holding capacity of yogurt gels, as calculated by analysis of variance (*n* = 3).

Significance (*p* Values)					
Contrast	Firmness	Rupture Force	Adhesiveness	Relaxation	WHC
Model	<0.0001	0.0004	<0.0001	<0.0001	0.5114
Substitution Level (A)	<0.0001	<0.0001	<0.0001	<0.0001	0.4348
Time (B)	0.9781	0.2021	0.0604	0.7538	0.3903
A × B	0.4579	0.7150	0.2156	0.1394	0.5124

**Table 2:** Summary of the significance of the effects of WBAP and time on the response of milk to heating, as calculated by analysis of variance (*n* = 3).

Significance (*p* Values)		
Contrasts	Particle Size *D*[4,3]	[-SH]_free_
Model	0.0044	<0.0001
Level of substitution (A)	0.0002	<0.0001
Time (B)	0.8507	0.0174
A × B	0.6332	0.1394

## 3. Results and Discussion

Heat-denatured whey protein can reinforce the yogurt gel matrix, but is also a major contributor to increased gel water-holding capacity, firmness, and apparent/complex viscosity [23,24]. Previous observations indicate decreased firmness in yogurts made with MWP compared to controls [6,7,8]. However, Britten and Giroux showed that the heat denaturing of the proteins can be adjusted in order to control the texture and syneresis of yogurt [25].

### 3.1. Whey:Buttermilk Protein Aggregate Composition and Properties

The composition of the dried dairy products is given in Table 3. The protein content of WBAP was 57.3% ± 0.1%, compared to 35.8% ± 0.1% for powdered skim milk. Powdered whey permeate was used to increase total solids, due to its high lactose content (85.9% ± 3.1%) and low protein content (2.2% ± 0.1%). The fat content of WBAP was 12.7% ± 0.3%. We hypothesized that this content made little difference because of the homogenization step (69 MPa) during the preparation of the powder. Our previous results showed that 25% whey/75% buttermilk based on protein mass gave the least hydrated aggregates. The water-holding capacity of WBAP aggregates was calculated at 2.04 ± 0.18 g_water_/g_protein_, and the percent of non-soluble protein at 78.5% ± 3.4%. 

**Table 3:** Composition of heat-denatured whey:buttermilk aggregate (WBAP), whey permeate (WP), and powdered skim milk.

	% Dry Matter			
	Protein	Lactose	Fat	Ash
WBAP	57.3 ± 0.1	18.8 ± 0.0	12.7 ± 0.3	4.9 ± 0.0
WP	2.2 ± 0.1	85.9 ± 3.1	0.2 ± 0.3	6.2 ± 0.0
Skim milk	35.8 ± 0.1	56.6 ± 3.4	1.0 ± 0.3	8.0 ± 0.0

The particle size distribution of whey:buttermilk aggregate during preparation (before homogenization) varied between 0.8 μm and 700 μm with an average of 50.5 μm (results not shown). Lelièvre concluded that incorporation of larger heat-denatured whey protein particles during the cheese-making process interferes with the casein network [2]. To facilitate particle retention and minimize product defects, aggregate particle size distribution should range between 0.1 μm and 10 μm. Homogenizing at a pressure of 69 MPa prior to freeze-drying reduced WBAP particle size to an average of 2.7 μm (results not shown). 

### 3.2. Particle Size in Skim Milk

**Figure 1:**
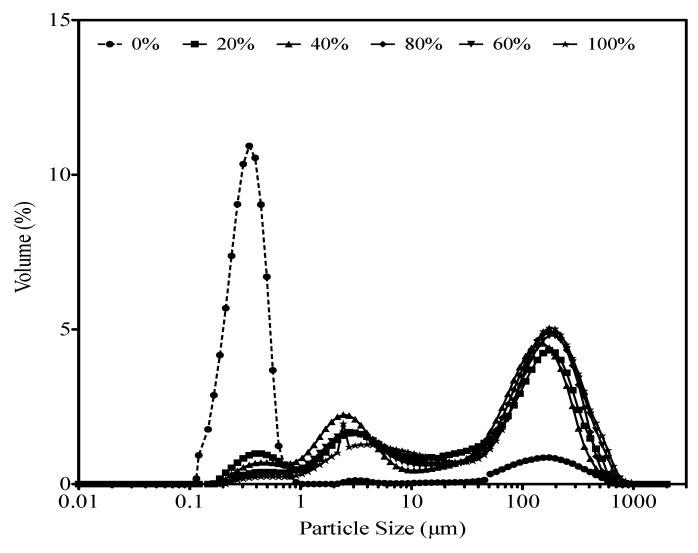
Particle size distribution profile in skim milk with the added powdered skim milk substituted with WBAP.

Particle size distribution in enriched skim milk containing various levels of WBAP replacing added powdered skim milk is shown in Figure 1. As shown in Table 2, only the substitution level had a significant effect on particle size (*p* = 0.0002). Average size was 22.9 ± 19.1 μm for skim milk and 159.3 ± 36.5 μm, 90.5 ± 42.8 μm, 129.6 ± 61.6 μm, 114.7 ± 27.4 μm, and 170.7 ± 50.9 μm respectively for 20%, 40%, 60%, 80%, and 100% of substitution of powdered skim milk by WBAP (results not shown). Comparison of the means indicates that average particle size was lower in skim milk than in skim milk + WBAP.

Figure 1 show that the particles were divided into two different populations in the skim milk corresponding first to casein micelle particles with an average diameter of 0.2 µm and to large particles with an average diameter of 200 µm. In the skim milk with the added powdered skim milk substituted, a third peak corresponding probably to WBAP particles (average diameter of 3 µm) can be observed. The more important proportion of higher average size particles in the mixtures could be attributed to possible interactions between skim milk proteins and aggregates present in WBAP. To the best of our knowledge, no conclusion has been reached in the literature about the properties of heat-denatured milk protein aggregates, and particularly their ability to participate in subsequent formation of heat-induced aggregates. Some researchers have concluded that added heat-denatured whey proteins are incorporated into other protein matrices, but this idea remains a hypothesis [6,7]. The present results suggest that some of the added heat-denatured whey proteins do interact with milk proteins during heating. Another plausible explanation would be that WBAP is less dispersible than skim milk protein, especially after only 30 min of stirring.

### 3.3. Exposure of Thiol Groups

**Figure 2:**
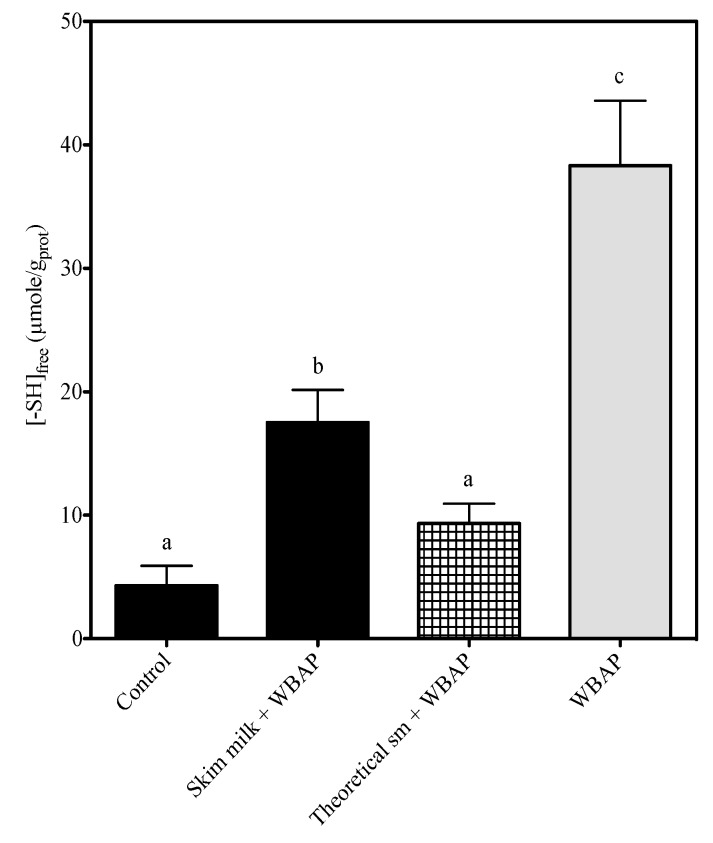
Effect of milk composition on thiol group exposure following heating (85 °C for 25 min, including ramp time); a Duncan post-test was applied to compare the means to the control.

Exposed thiol group concentration as a function of milk composition is presented in Figure 2. As shown in Table 2, both the level of substitution (*p* < 0.0001) and the heating time (*p* = 0.0174) had a significant effect on the exposure of thiol groups. The concentration was 4.3 ± 1.6 µmol/g_prot_ in milk enriched with powdered skim milk. When heated alone in water, WBAP had 38.3 ± 5.3 µmol of exposed thiol groups per g of protein. The concentration in the skim milk + WBAP mixture (17.5 ± 2.6 µmol/g_prot_) was unexpectedly higher than the calculated theoretical concentrations, 9.3 ± 1.6. The calculated theoretical concentrations were not significantly different from the level measured in the control skim milk.

These results suggest that re-suspended WBAP aggregates could interact with skim milk proteins through thiol/disulfide exchanges. 

### 3.4. Distribution of Particles in Enriched Skim Milk before Acidification

**Figure 3:**
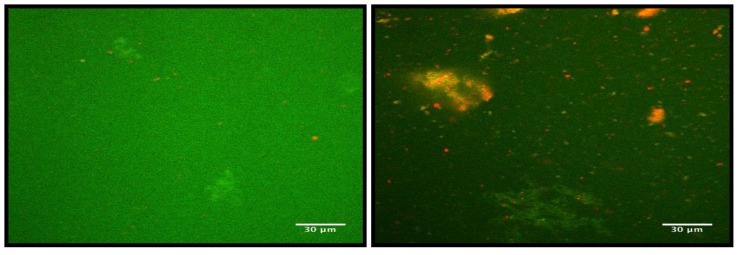
Images of control (enriched with powdered skim milk) skim milk (left) and skim milk + WBAP aggregates (0.6 g/100 mL; right) at pH 6.8, using the Nikon C1 confocal laser scanning microscope at 60×; proteins are dyed green and phospholipids are red.

Confocal laser scanning microscope images of enriched skim milk heated to 100 °C, mixed with glucano-delta-lactone and stained are presented in Figure 3. These show that protein aggregates (diameter ~ 30 μm) after heating were fewer in milk enriched with powdered skim milk than in milk enriched with WBAP, which contained larger aggregates and many small aggregates. The amount of fat was also greater in the WBAP-enriched milk.

### 3.5. Texture of Yogurts

#### 3.5.1. Appearance of Yogurts

Lucey and Singh defined the appearance of set-type gel as smooth with no cracks and no surface whey [26]. Control yogurts and those enriched with WBAP both had a smooth consistency with no whey separation. All gels were consistent and free of cracks or holes. At all levels of substitution of powdered skim milk with WBAP, a slight sedimentation of particles was observed. At 100% substitution, the gels were grainy. Schmidt *et al.* [27] attributed the grainy texture to the heat treatment, and particularly to treatments at 90 °C for 30 min. Since our yogurt-making process involved heating to 85 °C, these defects are likely due to insufficient hydration time (30 min) for the denatured proteins in the added WBAP.

#### 3.5.2. Firmness

**Figure 4:**
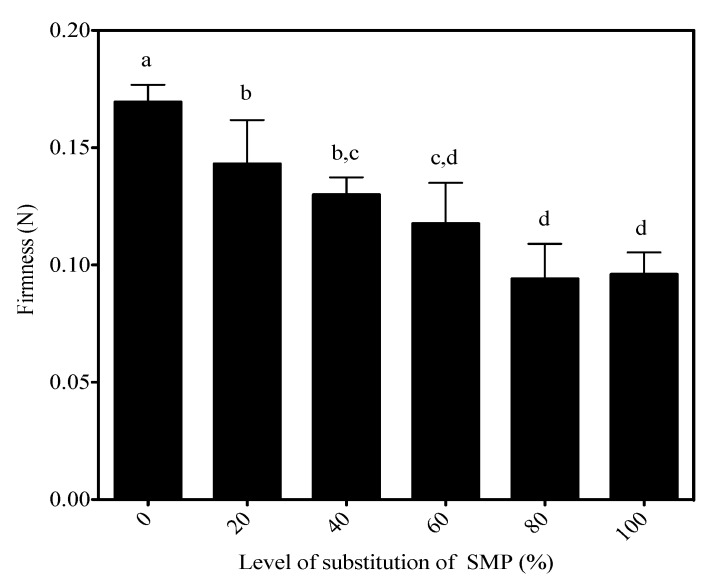
Firmness of control set-type yogurt, and yogurts enriched with whey:buttermilk protein aggregates. A Duncan post-test was applied to compare the means to the control.

The effect of WBAP as a substitute for powdered skim milk on the firmness of the acid-set type yogurt gels is presented in Figure 4. Table 1 shows that only the substitution factor was significant (*p* < 0.0001). The firmness was 0.170 ± 0.007 N for yogurts made from milk enriched with powdered skim milk, and decreased from 0.143 ± 0.019 N at 20% substitution to 0.096 ± 0.009 N at 100% substitution. Comparison of means shows that the firmness resulting from 0% substitution was significantly higher than was obtained even in the case of low levels of substitution.

We believe that the decrease in firmness was due mainly to the post-heating particle size. During gelation, casein chains shrink, and the density of the matrix increases by reduction of the pore dimension [23]. It can be hypothesized that the presence of large aggregates prevented the contraction of the casein network, and consequently weakened the gel at certain points. This idea is consistent with the trend of the particle distribution in skim milk and enriched skim milk. 

#### 3.5.3. Rupture Force, Adhesiveness, and Relaxation

The effect of WBAP as a substitute for powdered skim milk on the rupture force, adhesiveness, and relaxation of the acid-set type gels is shown in Table 4.

Table 1 again shows that only the substitution factor was significant (*p* < 0.0001). The rupture force was 0.093 ± 0.005 N for yogurts made from milk enriched with powdered skim milk, and decreased from 0.072 ± 0.010 at 20% substitution to 0.051 ± 0.009 N at 100% substitution. The comparison of means showed that the yogurts made using enrichment with WBAP were more susceptible to rupture than were those made using enrichment with powdered skim milk only. However, no significant difference was observed between 20%, 40% and 60% substitution. Gel relaxation was 60.2% ± 0.6% for yogurts made from milk enriched with powdered skim milk, and decreased from 56.9% ± 2.5% at 20% substitution to 47.2% ± 2.7 at 100% substitution. Comparison of means shows that the relaxation resulting from 0% substitution did not differ significantly from that obtained with 20% or 40% substitution.

**Table 4:** Summary of the mean rupture force, adhesiveness and relaxation of yogurt gels, as a function of percent substitution of powdered skim milk with whey:buttermilk aggregate enriching the starting milk. A Duncan post-test was applied to compare the means to the control.

Level of Substitution (%)	Rupture Force (N)	Adhesiveness (N.s)	Relaxation (%)
0	0.093 ^a^ ± 0.005	−0.614 ^a^ ± 0.083	60.2 ^a^ ± 0.6
20	0.072 ^b^ ± 0.010	−0.374 ^b^ ± 0.190	56.9 ^a,b^ ± 2.5
40	0.067 ^b^ ± 0.005	−0.274 ^b,c^ ± 0.071	55.7 ^a,b^ ± 1.8
60	0.067 ^b^ ± 0.005	−0.239 ^b,c^ ± 0.098	52.3 ^b,c ^ ± 3.9
80	0.057 ^c^ ± 0.011	−0.105 ^c^ ± 0.033	49.8 ^c,d ^ ± 2.7
100	0.051 ^c^ ± 0.009	−0.094 ^c^ ± 0.032	47.2 ^d^ ± 2.7

Means within a column sharing the same letter are not significantly different (*p* > 0.05).

Adhesiveness was −0.614 ± 0.083 N.s for yogurts made from milk enriched with powdered skim milk, and decreased from −0.374 ± 0.190 N.s at 20% substitution to −0.094 ± 0.032 N.s at 100% substitution with WBAP. Comparison of means shows that increasing the level of substitution decreased the tendency of the gels to adhere to the probe surface because the absolute value of the area is higher. Similar results were obtained using fat replacers [7].

### 3.6. Water-Holding Capacity of Yogurt Gels

**Figure 5:**
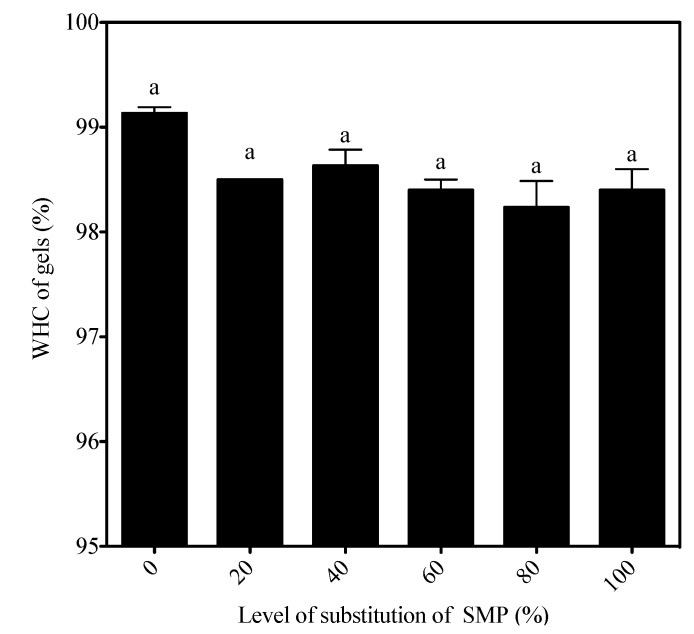
Water-holding capacity of set-type yogurt, and yogurts made from milk enriched with whey:buttermilk protein aggregates.

The effect of substituting powdered skim milk with WBAP on the water-holding capacity (WHC) of the yogurt gels is shown in Figure 5. Table 2 shows no significant differences (*p* = 0.5114) between the samples. The WHC was 99.1% ± 0.1% for yogurt made from milk enriched with powdered skim milk, and varied from 98.6% ± 0.1% to 98.2% ± 0.2% in yogurts containing WBAP.

Gel water-holding capacity is defined as the resistance of the gel to compaction (if force>500 g) or to whey separation (if force<500 g). Centrifugation parameters (temperature, time, force) differ from one study to the next. However, it is an accepted view that heating the milk increases the water-holding capacity of the resulting set-type yogurt [23]. Adding large amounts of denatured whey protein led to the formation of a network composed of casein micelle chains able to immobilize large amounts of water [28]. It was later confirmed that adding more whey protein before heating led to increased immobilization of free water in the yogurt gel, due mainly to increased gel compactness [29]. By replacing skim milk proteins with pre-formed aggregates, the amount of denatured protein in the system was changed. This could explain the small (not significant) decrease in the water-holding capacity of the gels. The textural properties of the gels showed that the size of the added aggregates affects the strength and the distribution of the protein network. Large pores allow a more open structure, facilitating the expulsion of water.

### 3.7. Simulation of the Gel Formation

**Figure 6:**
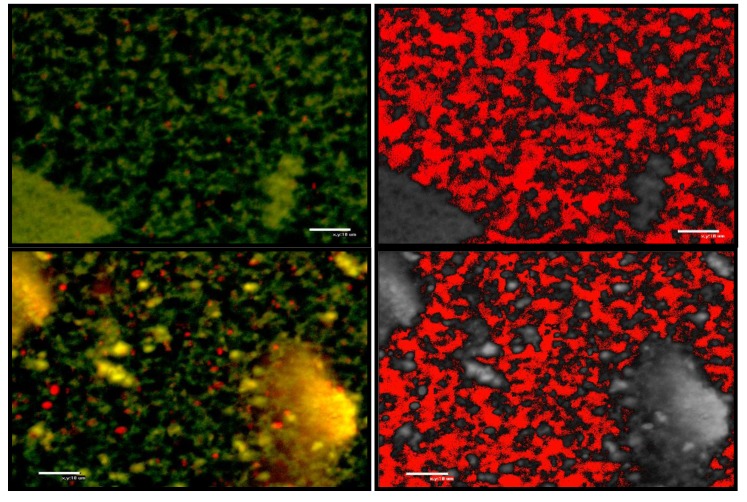
Confocal laser scanning microscope (Nikon C1) images taken of gels of control skim milk (top) and skim milk + WBAP (0.6 g/100 mL; bottom) at 60× with a zoom of 2.31×; proteins were dyed green and phospholipids are red. Images on the right were processed with ImageJ to color the clusters red. Scale bar value = 30 µm.

Confocal micrographic images taken when gel formation in milk was completed are presented in Figure 6. The structure of the gel formed when the milk was enriched with powdered skim milk featuring small protein aggregates linked together. Heating led to the formation of large aggregates, and fat globules were entrapped in the matrix. The images transformed via ImageJ show that the clusters were homogenous in size, and distributed evenly throughout the gel structure. The structure of the WBAP-enriched milk gel featured small and large protein aggregates linked together. Fat globules were also entrapped in the gel. The presence of yellow/orange particles suggests that the structure of these aggregates could include both protein and fat. The size of the clusters was less homogenous than in the gel made from milk enriched with powdered skim milk. Small clusters were present around the large aggregates, and large clusters were present around small aggregates. 

**Figure 7:**
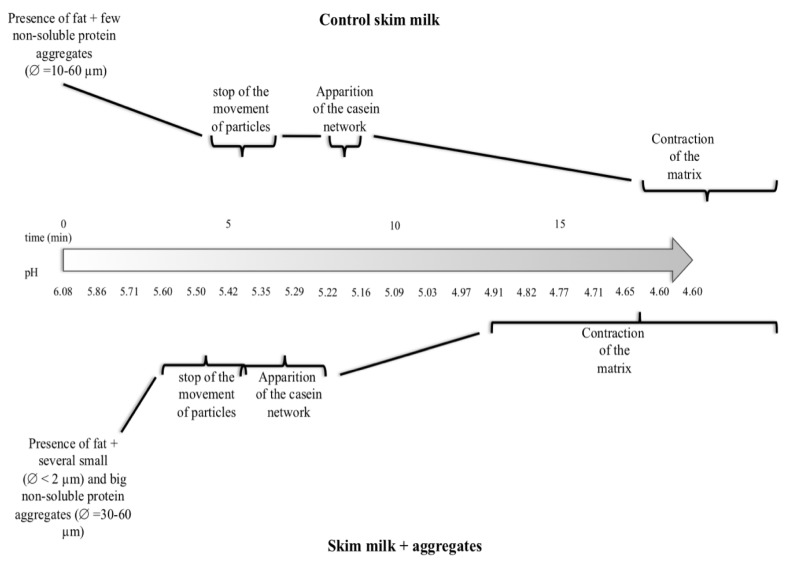
General trends observed in the videos of gel formation as a function of pH and time.

Figure 7 summarizes the general trends observed in videos of gel formation as a function of pH and time in milk enriched with powdered skim milk or WBAP. As noted above, few large particles remained in the case of powdered skim milk after the heat treatment. However, all particles were moving for the first approximately 5 min (pH ranged from 5.50 to 5.29). The movement was intense, meaning that the initially observed particles moved quickly out of the field of view and were replaced by other particles. The first gel structure appeared about one minute after immobilization of the particles (pH ranged from ~5.22 to 5.16). The structure of the gel became more apparent as the pH decreased. The matrix started to contract around pH 4.65.

The trend observed was slightly different in the WBAP-milk. As noted above, numerous large particles and many small ones were present after heating. Unlike in the case of powdered skim milk enrichment, these large particles were not very mobile. Small particles were mobile but the flow was slower than in the powdered skim milk case. Particle movement also stopped sooner (at pH ~5.60), and the first gel structure appeared almost instantaneously. The matrix started to contract when the pH reached 4.91. For no obvious reason, emission by the fluorophores (especially Oregon 488) decreased rapidly, and the images ended up dark. However, it was still possible to see the gel using ImageJ software.

## 4. Conclusions

The present study shows that heat-denatured whey:buttermilk protein aggregates behaves as active fillers in yogurt. Moreover, heated skim milk proteins including whey proteins and caseins may interact with WBPA because of their high thiol group content. Replacing powdered skim milk with whey:buttermilk aggregate as an enrichment for milk used for yogurt production significantly affected certain textural properties of the gels. Fracture force, firmness, and relaxation decreased as the level of substitution increased, while adhesive force increased. The water-holding capacity of yogurt gels is slightly but not significantly affected. Overall, the results show that it is possible to use heat-denatured whey:buttermilk protein aggregate in the production of set-type yogurt, but the dispersibility of the powdered aggregate must be strictly controlled in order to limit the presence of large particles. 
